# Spontaneous Pneumomediastinum Following Severe Vomiting in a Previously Healthy Young Adult

**DOI:** 10.7759/cureus.102388

**Published:** 2026-01-27

**Authors:** Anas E Ahmed, Kareman O Abdulhadi, Abdulkarim S Alofi, Mohammed A Binhussain, Ahmed H AlMohammedsaleh

**Affiliations:** 1 Community Medicine, Jazan University, Jazan, SAU; 2 College of Medicine, Batterjee Medical College, Jeddah, SAU; 3 College of Medicine, First Moscow State Medical University, Moscow, RUS; 4 College of Medicine, King Faisal University, Al-Ahsa, SAU

**Keywords:** chest pain, computed tomography, conservative management, dyspnea, esophageal perforation differential diagnosis, macklin effect, severe vomiting, spontaneous pneumomediastinum, subcutaneous emphysema

## Abstract

Spontaneous pneumomediastinum is an uncommon and generally benign condition that can present with acute chest pain and respiratory symptoms, often mimicking life-threatening cardiopulmonary or esophageal emergencies. Although frequently associated with asthma or coughing, severe vomiting is a less commonly recognized precipitating factor and poses a particular diagnostic challenge due to its overlap with esophageal perforation. We report the case of a previously healthy young adult who developed spontaneous pneumomediastinum following repeated episodes of forceful vomiting, presenting with retrosternal chest pain, dyspnea, subcutaneous emphysema, and a characteristic precordial crunch. Imaging studies confirmed the presence of mediastinal air without evidence of secondary causes, supporting a diagnosis consistent with alveolar rupture from increased intrathoracic pressure. The patient was managed conservatively with supportive care, resulting in complete clinical and radiologic resolution. This case underscores the importance of recognizing vomiting-induced spontaneous pneumomediastinum, maintaining a structured diagnostic approach to exclude more serious conditions, and avoiding unnecessary invasive interventions in clinically stable patients.

## Introduction

Spontaneous pneumomediastinum is an uncommon clinical entity characterized by the presence of free air within the mediastinal space in the absence of traumatic, iatrogenic, or other identifiable secondary causes [1,2]. It predominantly affects young adults and is generally considered a benign and self-limiting condition [2,3]. The pathophysiological mechanism most frequently implicated is the Macklin effect, in which a sudden increase in intra-alveolar pressure leads to alveolar rupture with subsequent dissection of air along the bronchovascular sheaths into the mediastinum [1-4]. Various precipitating factors have been described, including asthma exacerbations, vigorous coughing, intense physical exertion, childbirth, and forceful vomiting. Due to its rarity and often nonspecific presentation, spontaneous pneumomediastinum may pose a diagnostic challenge and can be mistaken for more serious cardiopulmonary or esophageal conditions [2,3].

Severe vomiting is a recognized but less frequently emphasized trigger of spontaneous pneumomediastinum and warrants particular attention because of its clinical overlap with life-threatening etiologies such as esophageal perforation [1,5]. Patients commonly present with acute chest pain, dyspnea, and subcutaneous emphysema, symptoms that necessitate thorough evaluation to exclude secondary causes. Imaging studies, particularly chest radiography and computed tomography, play a crucial role in establishing the diagnosis and guiding management [1-4]. Awareness of this condition and its distinguishing features is essential to avoid unnecessary invasive interventions and to ensure appropriate conservative treatment. This report describes a case of spontaneous pneumomediastinum following severe vomiting, highlighting its clinical presentation, diagnostic work-up, and favorable outcome with supportive management.

## Case presentation

A previously healthy young adult presented to the emergency department with acute-onset retrosternal chest pain and shortness of breath following multiple episodes of forceful vomiting. The patient reported a 24-hour history of severe, repetitive non-bilious, non-bloody emesis preceded by nausea after presumed acute gastroenteritis. There was no history of recent trauma, endoscopic procedures, vigorous coughing, asthma exacerbation, illicit drug use, or intense physical exertion. The patient denied dysphagia, odynophagia, hematemesis, fever, or prior similar episodes. Past medical and surgical history was unremarkable, with no known chronic pulmonary disease. The patient was a non-smoker and denied alcohol binge or recreational drug use. There was no significant family history of connective tissue disorders or spontaneous air-leak syndromes.

On initial assessment, the patient was alert and oriented but appeared uncomfortable due to chest pain. Vital signs revealed mild tachycardia with a heart rate of 104 beats per minute, a blood pressure of 118/72 millimeters of mercury, a respiratory rate of 22 breaths per minute, an oxygen saturation of 97% on room air, and an afebrile temperature. Physical examination of the chest demonstrated mild tachypnea without the use of accessory muscles. Palpation revealed subtle crepitus over the supraclavicular region and anterior chest wall, suggestive of subcutaneous emphysema. Auscultation of the lungs revealed normal breath sounds bilaterally without wheezes or crackles. A faint precordial crunch synchronous with the cardiac cycle was appreciated, consistent with Hamman's sign. Cardiovascular examination was otherwise normal. Abdominal examination showed mild epigastric tenderness without guarding or rigidity. Neurological and musculoskeletal examinations were unremarkable.

Initial laboratory investigations demonstrated a mild leukocytosis with a white blood cell count of 12.4×10⁹ per liter, likely reactive. Hemoglobin, platelet count, and basic metabolic panel were within normal limits. Serum electrolytes showed mild hypokalemia attributed to vomiting. Arterial blood gas analysis on room air revealed no hypoxemia or acid-base disturbance. Cardiac biomarkers, including high-sensitivity troponin, were negative. Inflammatory markers were not significantly elevated. Serum amylase and lipase levels were within normal range, reducing suspicion for pancreatitis.

**Figure 1:**
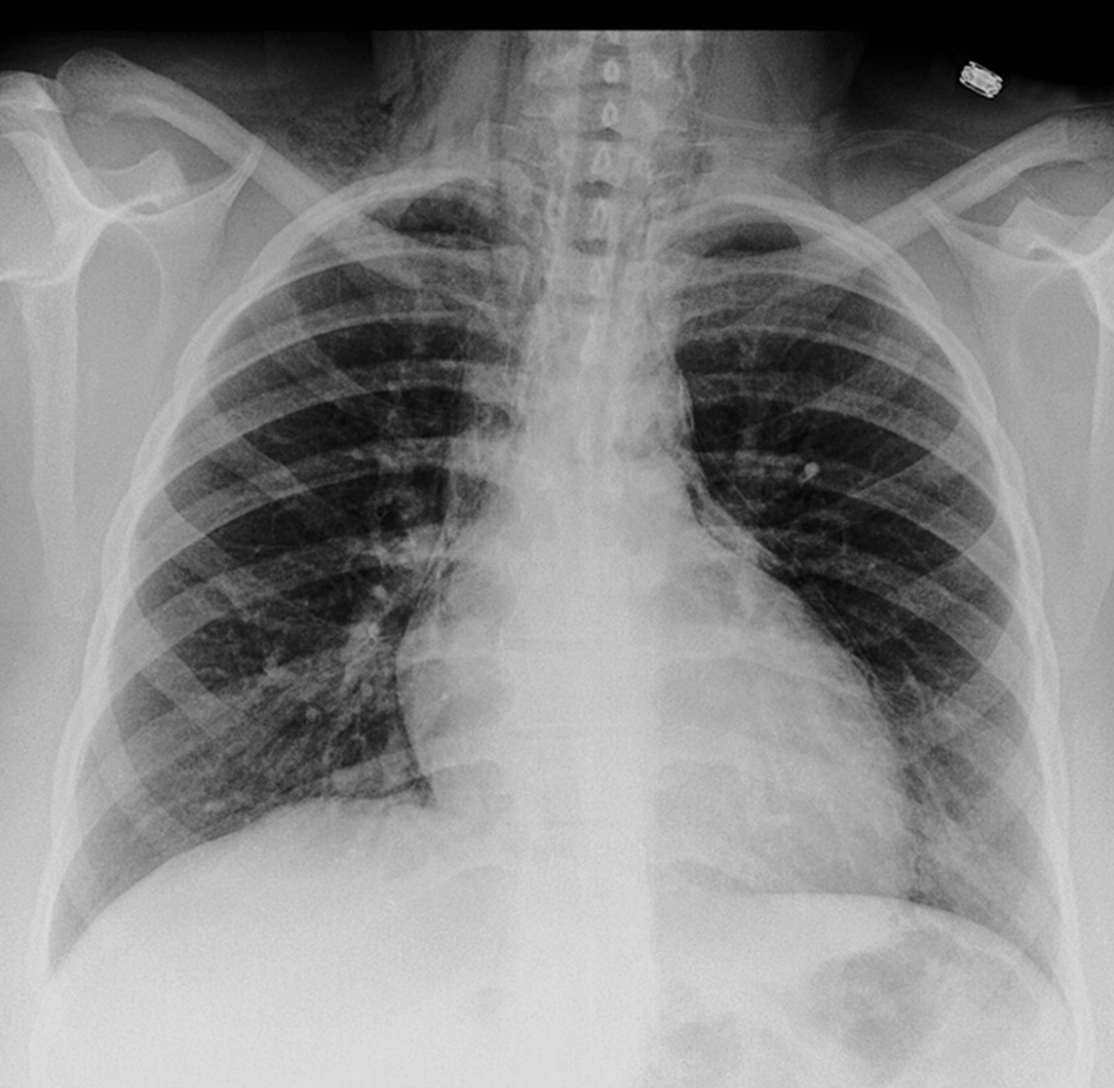
Posteroanterior chest radiograph demonstrating pneumomediastinum, evidenced by thin streaks of air outlining the cardiac silhouette and superior mediastinal structures

**Figure 2:**
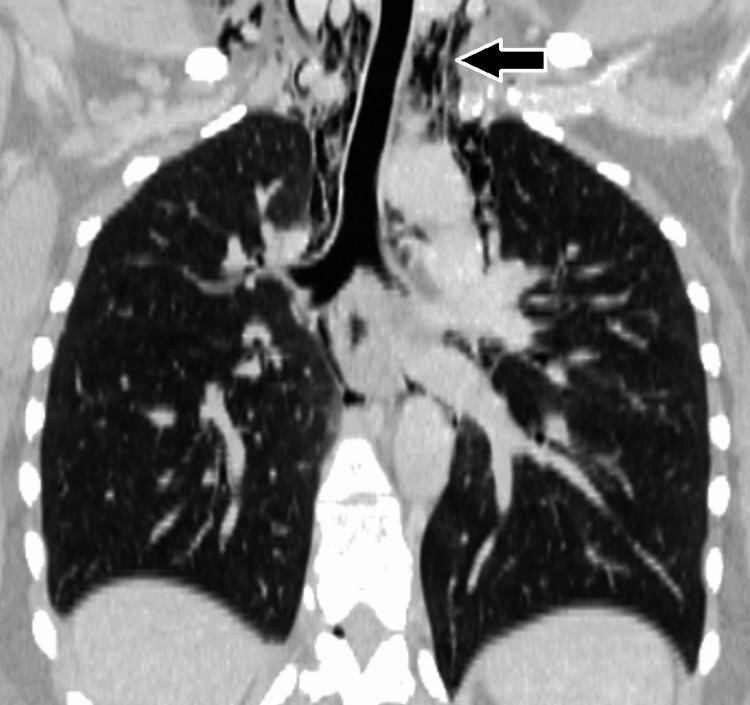
Coronal reformatted computed tomography image of the chest confirming the presence of extraluminal air (arrow) within the superior mediastinum, adjacent to the trachea

A posteroanterior and lateral chest radiograph demonstrated lucent streaks of air outlining the mediastinal contours, particularly along the cardiac silhouette and great vessels, consistent with pneumomediastinum (Figure 1). There was associated subcutaneous emphysema in the cervical region. No evidence of pneumothorax or pleural effusion was identified. To further characterize the findings and exclude secondary causes, a contrast-enhanced computed tomography scan of the chest was performed. Computed tomography imaging confirmed extensive free air within the mediastinum extending into the cervical soft tissues without evidence of esophageal perforation, tracheobronchial injury, or pneumothorax (Figure 2). The lung parenchyma appeared normal with no bullae or interstitial disease. An oral contrast esophagogram was not deemed necessary given the absence of clinical signs of esophageal rupture and reassuring computed tomography findings.

The differential diagnosis included Boerhaave syndrome, acute coronary syndrome, pulmonary embolism, pericarditis, pneumothorax, and asthma-related barotrauma. Boerhaave syndrome was carefully considered given the history of forceful vomiting; however, the absence of severe systemic toxicity, mediastinal fluid collections, pleural effusion, or contrast leak on computed tomography made this diagnosis unlikely. Cardiac and thromboembolic causes were excluded based on clinical assessment, laboratory testing, and imaging. The overall clinical and radiologic picture supported a diagnosis of spontaneous pneumomediastinum secondary to increased intra-alveolar pressure from severe vomiting, consistent with the Macklin effect.

The patient was admitted for observation and conservative management. Treatment consisted of supplemental oxygen to facilitate nitrogen washout, intravenous fluids for hydration, antiemetic therapy, and analgesia with non-opioid medications. Oral intake was initially withheld and gradually resumed as symptoms improved. Prophylactic antibiotics were not administered due to a lack of evidence of infection or esophageal injury. The patient remained hemodynamically stable throughout hospitalization, with progressive improvement in chest pain and dyspnea. Serial physical examinations demonstrated resolution of subcutaneous emphysema, and repeat chest radiography after 48 hours showed a marked reduction of mediastinal air. The patient was discharged on hospital day 3 in stable condition with instructions for activity restriction, avoidance of Valsalva-inducing maneuvers, and outpatient follow-up. At a two-week follow-up visit, the patient reported complete resolution of symptoms.

## Discussion

Spontaneous pneumomediastinum is an infrequent but well-recognized clinical entity defined by the presence of mediastinal free air without an identifiable traumatic, iatrogenic, or secondary pathological cause [1-6]. Although generally benign, its presentation often mimics life-threatening conditions, making timely recognition and appropriate management essential [2,3]. The present case highlights severe vomiting as a precipitating factor for spontaneous pneumomediastinum and underscores the importance of differentiating this condition from esophageal perforation and other critical cardiopulmonary emergencies.

The pathophysiology of spontaneous pneumomediastinum is most commonly explained by the Macklin effect, in which a sudden increase in intra-alveolar pressure leads to alveolar rupture, allowing air to dissect along the bronchovascular sheaths toward the mediastinum [2,4]. Forceful vomiting can generate significant intrathoracic pressure through repeated Valsalva maneuvers, predisposing to alveolar rupture even in the absence of underlying lung disease [2-4]. While asthma and coughing are more frequently reported triggers, vomiting-induced spontaneous pneumomediastinum is likely underrecognized and may be overlooked due to concern for Boerhaave syndrome, a far more serious condition associated with high morbidity and mortality if missed [2-4].

Clinically, SPM often presents with acute retrosternal chest pain, dyspnea, neck pain, or dysphonia and may be accompanied by subcutaneous emphysema or Hamman's sign. However, these findings are neither universally present nor specific [1-6]. As in the current case, patients may be hemodynamically stable with minimal respiratory compromise, which should raise suspicion for a benign etiology. Nevertheless, given the overlap in presentation with acute coronary syndrome, pulmonary embolism, pneumothorax, and esophageal rupture, a systematic diagnostic approach is mandatory. Laboratory investigations are typically nonspecific and mainly serve to exclude alternative diagnoses or identify complications such as infection or electrolyte disturbances secondary to vomiting [2-8].

Imaging remains central to the diagnosis of spontaneous pneumomediastinum [5-8]. Chest radiography is often sufficient for initial detection, demonstrating lucent streaks of mediastinal air or subcutaneous emphysema. However, computed tomography of the chest is considered the diagnostic gold standard, offering superior sensitivity and allowing exclusion of secondary causes, including tracheobronchial or esophageal injury [3,4]. In patients with vomiting-induced spontaneous pneumomediastinum, computed tomography findings such as absence of mediastinal fluid collections, pleural effusions, or contrast extravasation are particularly reassuring [1,4]. Routine use of invasive diagnostic modalities, including esophagography or endoscopy, is increasingly discouraged in clinically stable patients with negative computed tomography findings, as these investigations rarely alter management and may expose patients to unnecessary risk [5,6].

Management of spontaneous pneumomediastinum is predominantly conservative, consisting of oxygen therapy, analgesia, rest, and treatment of the underlying precipitating factor [3,5]. Supplemental oxygen is thought to accelerate reabsorption of mediastinal air by increasing the diffusion gradient for nitrogen, although high-quality evidence is limited [1-7]. Antibiotics and dietary restriction are not routinely indicated unless there is a strong suspicion of esophageal injury or infection. The favorable clinical course observed in this case aligns with existing literature, which consistently reports excellent outcomes and low recurrence rates with supportive care alone [3,5,8].

Hospitalization duration and need for monitoring remain topics of debate. While some authors advocate for brief observation or even outpatient management in selected patients, admission remains reasonable for initial monitoring, particularly when the diagnosis is uncertain or symptoms are significant [1-7]. Importantly, recurrence of spontaneous pneumomediastinum is rare, and long-term sequelae are uncommon. Patient education regarding avoidance of precipitating activities and prompt management of vomiting or coughing episodes is a key component of follow-up care.

## Conclusions

Spontaneous pneumomediastinum should be considered in patients presenting with acute chest pain and dyspnea following episodes of severe vomiting, particularly when clinical stability and benign examination findings are present. Although uncommon, this condition closely mimics more serious etiologies such as esophageal perforation and cardiopulmonary emergencies, making a systematic diagnostic approach essential. Chest computed tomography plays a pivotal role in confirming the diagnosis and safely excluding secondary causes. Once identified, spontaneous pneumomediastinum can be effectively managed with conservative supportive measures, resulting in rapid symptom resolution and excellent outcomes. Early recognition of this entity helps prevent unnecessary invasive investigations and interventions, underscoring the importance of clinical awareness and appropriate imaging in achieving optimal patient care.
